# Translation and Validation of the Risk Perception on Safety and Health Questionnaire Among Palm Oil Plantation Workers in Terengganu, Malaysia

**DOI:** 10.7759/cureus.44742

**Published:** 2023-09-05

**Authors:** Syahrul Faiz Zakaria, Mohd Nazri Shafei, Wan Nor Arifin

**Affiliations:** 1 Community Medicine, School of Medical Sciences, Universiti Sains Malaysia, Kubang Kerian, MYS; 2 Biostatistics and Research Methodology, School of Medical Sciences, Universiti Sains Malaysia, Kubang Kerian, MYS

**Keywords:** workplace safety, translation and validation, palm oil plantation workers, occupational safety and health, risk perception

## Abstract

Introduction: Occupational safety and health are of utmost importance in the palm oil industry, particularly in Malaysia where palm oil plantation workers face various hazards. This study aimed to translate and validate a risk perception questionnaire specifically designed to assess the perceptions of safety and health risks among palm oil plantation workers in Terengganu, Malaysia.

Methodology: The original risk perception questionnaire, consisting of 22 items, was translated into the local language and culturally adapted. The translation process involved forward translation, expert panel discussions, and back translation to ensure linguistic equivalence. The translated questionnaire was then administered to a sample of palm oil plantation workers in Terengganu, Malaysia, for validation purposes. Confirmatory factor analysis (CFA) was conducted to assess the model fitness of the questionnaire, and Cronbach's Alpha coefficient was calculated to determine the internal consistency reliability of the final model.

Results: The translated risk perception questionnaire demonstrated good model fitness as indicated by CFA results (X^2^=224, df=79, p-value<0.0001, root mean square error of approximation (RMSEA)=0.07, goodness of fit index (GFI)=0.929, comparative fit index (CFI)=0.902). The questionnaire's final version has one factor comprising 13 items, selected based on factor loadings and theoretical relevance. The internal consistency reliability of the 13-item questionnaire was satisfactory, with a Cronbach's Alpha coefficient of α=0.77.

Conclusion: The 13-item risk perception questionnaire demonstrated a good model fit and acceptable internal consistency reliability. It shows that the questionnaire is a valid and reliable tool to evaluate the level of risk perception on safety and health among palm oil plantation workers in Malaysia.

## Introduction

The agricultural industry plays a vital role in economies worldwide by supplying food and essential resources to local and global markets. However, like any field, it presents unique occupational risks that endanger the health and safety of workers [1]. These hazards vary depending on the agricultural type, location, and specific tasks involved. Common hazards in agriculture include exposure to pesticides and chemicals, physical injuries from machinery, equipment-related accidents, and musculoskeletal disorders (MSDs) resulting from repetitive manual labor [2]. Furthermore, working in extreme weather conditions or near livestock can also pose risks. These hazards can lead to various health issues, ranging from immediate injuries to long-term illnesses, and can significantly impact workers' livelihoods and well-being. Therefore, it is crucial for workers, employers, and policymakers to acknowledge these risks and take necessary measures to prevent or mitigate their effects on agricultural workers worldwide. The agricultural sector is characterized by its diversity and complexity, involving various activities and practices. Despite the variations, those workers across the globe encounter similar risks and hazards [2].

Exposure to pesticides and chemicals also represents a significant hazard within the agricultural sector. Workers involved in handling or applying pesticides are particularly vulnerable to acute poisoning, which manifests in symptoms such as headaches, nausea, and respiratory issues. Prolonged exposure to pesticides has been associated with various health problems, including cancer, neurological disorders, and reproductive complications. Additionally, other chemicals utilized in agriculture, such as fertilizers and cleaning agents, can also pose risks to the workers' well-being [2].

Furthermore, physical injuries are prevalent in the agricultural industry, often stemming from the operation of heavy machinery and equipment. Accidents such as tractor rollovers, entanglement in machinery, and falls from elevated locations are among the numerous types of mishaps that can occur on farms and in similar agricultural environments. These injuries can be severe, leading to fatalities or long-term disabilities [1].

MSDs present a notable risk in the agricultural sector, especially for workers engaged in manual labor tasks like planting, harvesting, and packing crops [3,4]. These injuries arise from repetitive motions and uncomfortable body positions, potentially resulting in chronic pain and disability if not appropriately addressed.

Aside from MSDs, agricultural workers are also exposed to various other hazards. Working in extreme weather conditions poses risks, as does handling and caring for livestock. Furthermore, there is the potential for exposure to zoonotic diseases, which can be transmitted from animals to humans. These hazards can pose significant challenges for agricultural workers, particularly in low-income countries where access to protective equipment and medical care may be limited.

Despite the many risks and challenges faced by agricultural workers around the world, there are strategies and interventions that can help to mitigate these hazards. These may include training programs, protective equipment and clothing, improved equipment design, and policies and regulations that promote safe working conditions. By addressing the occupational hazards in the agricultural sector, we can help to ensure that workers in this important industry are able to work safely and maintain their health and well-being.

The agricultural sector is vital for sustaining economies worldwide, providing food security, and contributing to local and global markets [5,6]. Agricultural workers play a crucial role in these processes, facing various occupational risks and hazards inherent to their profession. Among these risks, understanding the perception of risk among agricultural workers is essential for promoting occupational health and safety practices [7,8]. By comprehending how agricultural workers perceive and assess risks, researchers and policymakers can develop targeted interventions and strategies to mitigate these risks effectively [9,10].

While research on risk perception in agriculture has gained attention in recent years, there is still a gap in the literature concerning the specific context of Malaysia. Malaysia has a diverse agricultural landscape, with a wide range of farming practices and crops cultivated. Despite its significance, the existing research on risk perception in this region is limited, particularly in relation to agricultural workers' perspectives [11].

To address this research gap, the present study aims to translate and validate the risk perception questionnaire from its original version in central Italy to the Malay language. This questionnaire was selected because it is one of the most comprehensive sets of questionnaires that cover all types of hazards in agriculture. As an example, the risk perception questionnaire that was validated by Soltani (2016) has many items (133 questions) and covers many parts that are not specific to hazards in agriculture like job situation and satisfaction, personal support, and help from others. Another tool to assess risk perception was used by Ali et al. (2019) to study the perception of farmers about their motivation to manage agricultural risk [11]. However, this six-item questionnaire with a Likert scale answers only focuses on the risk of climate change and other disasters on the farm but not working environment hazards that affect farmers. The translation and validation process of the risk perception questionnaire follows established guidelines to ensure the linguistic and psychometric equivalence of the translated version [12]. The translation process involves forward translation, expert panel discussions, and back translation to achieve conceptual and linguistic equivalence between the original and translated versions [13]. Subsequently, the translated questionnaire undergoes a validation process to assess its reliability and validity in measuring risk perception among agricultural workers [14].

## Materials and methods

Study design

This was a translation and validation study of a risk perception questionnaire. The translation and adaptation of the instrument from the original language into the Malay language were conducted. The Malay language is the Malaysian national language, and the main language conversed by Malaysians, especially among Federal Land Development Authority (FELDA) settlers most of whom are Malays. Next, a psychometric validation of the Malay-translated risk perception questionnaire was performed.

Based on Comfrey and Lee (1992), a minimum of 350 samples are considered good for a validation study [15]. After considering the 10% non-response rate, the minimum sample size required to achieve the objective was 385. The respondents were chosen by systematic random sampling from FELDA settlers all around Terengganu, Malaysia. The questionnaire was distributed and filled up by them.

The original risk perception questionnaire

The original risk perception questionnaire was obtained from a study by Cecchini et al. (2018) [16]. The consent to use the risk perception questionnaire was obtained from the original author before conducting the study. The structured questionnaire used in the study by Cecchini et al. (2018) was developed based on the theoretical framework of the Health Belief Model and the Theory of Planned Behavior. The questionnaire consisted of three sections which were (1) demographic information: this section included questions about age, gender, education level, years of experience in agriculture, and type of farm work; (2) safety knowledge: this section consisted of ten multiple-choice questions related to safe practices in agriculture, such as handling pesticides, using machinery, and preventing falls. Items in this section from the original questionnaire were added to the revised manuscript. This items include "Do you think that gloves are useful for vibration protection," "Do you think it is useful to adjust the seat with the appropriate lever or knob," and "Do you think the tractor can overturn when working on sloping ground;" (3) safety attitudes and behavior: this section included 23 questions that assessed attitudes toward safety in the workplace and self-reported safety behavior. The questions were rated on a five-point Likert scale ranging from "strongly disagree" to "strongly agree." Examples of questions include "I always wear protective equipment when I work on the farm" and "I think safety is an important part of my job.” The questionnaire was pretested on a sample of agricultural workers to ensure its validity and reliability. The Cronbach's alpha coefficient for the safety attitudes and behavior section was 0.87, indicating good internal consistency. 

Translation and cultural adaptation

**Figure 1:**
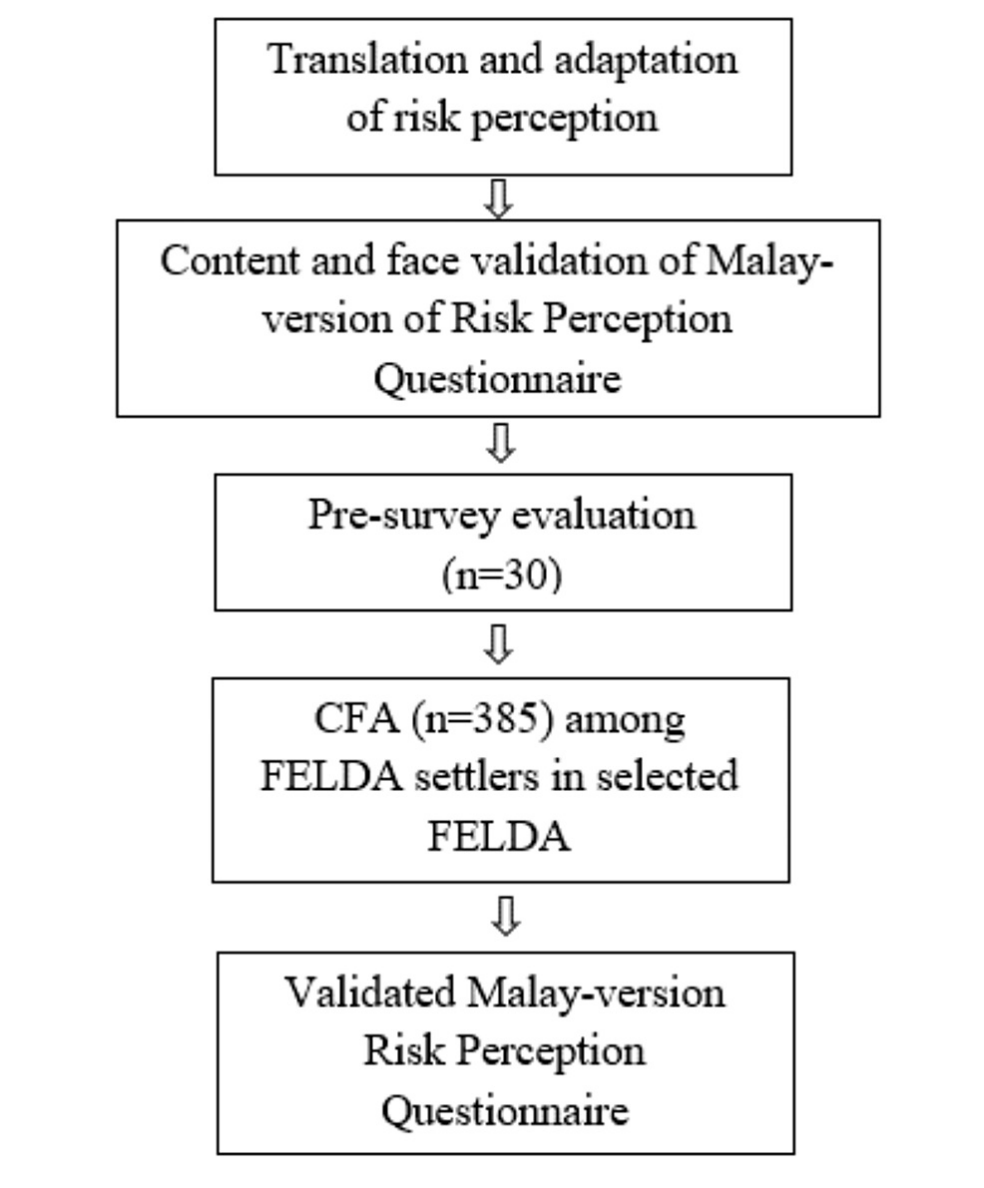
Study flowchart for translation and validation process

The process of translation and validation of the risk perception questionnaire is shown in Figure 1. To ensure the process of translation and cultural adaptation are standardized, guidelines endorsed by Wild et al. (2005) were followed [13]. This step-by-step guideline was made of the following ten stages.

Preparation

Before commencing with the translation, the initial stage was the preparation stage. This stage was meant to find the translator and set the deadlines for each stage.

Forward Translation

The forward translation stages hired three independent translators, a qualified professional translation service (Translator one), a teacher from the linguistic department in Universiti Sains Malaysia (Translator two), and a school teacher from Terengganu (Translator three). Translator one was assigned to translate the original questionnaire from the Italian language to both the English language and the Malay language. Translators two and three were native Malay speakers and conversed well and fluent in both English and Malay languages. Translator two is currently a professional translator with vast experience in translating medical background instruments.

All the translators conducted the translation from English to Malay language independently, and any difficult or confusing words, items, or terminologies were highlighted.

Reconciliation

The result of the three independent forward translations was compared and then merged and harmonized into a single translation. A committee of a safety and health expert, a public health specialist, a medical statistician, and medical doctors reviewed the single translation. Words, phrases, or terminologies that were confusing or irrelevant to the local context were substituted and adapted with more practical wording and phrases. In addition, content and face validity processes were also undertaken during this phase to further the concept and item adequacy.

Back Translation

After producing the reconciled single Malay translation, it was translated back to English to learn and compare with the original English version. Two back translators were hired and both shared similar criteria with translator two and translator three in the forward translation stage. A fortnight was allocated to produce the back translation.

Back Translation Review

Once the back translation was available, it was scrutinized for any discrepancies with the original English version by the same review committee. The committee assessed the equivalence, either conceptual, items, or semantic of both versions of the translation. Overall, the panels of the review committee consented to some minor adjustments to the back translation.

Harmonization

The harmonization stage meant to seek further agreement between the reconciled Malay-translated version and both the back-translated and original versions. This stage ran a quality control check to improve any undue discrepancies. It was concluded by the review committee that the reconciled Malay-translated version was opt to be the pre-final version of the Malay-translated risk perception questionnaire.

Cognitive Debriefing

Cognitive debriefing functioned as a test run for the pre-final version using a conveniently selected small group of respondents to test understandability or any adjustment required. Ten FELDA settlers were called to the FELDA Wilayah Terengganu headquarters office and engaged in this cognitive debriefing stage. All of them are initially briefed and then given the pre-final version of the Malay-translated risk perception questionnaire to be filled. From this session, there were a few difficult words to comprehend, and confusing terminologies were found. The respondents were asked for their opinions and recommendations to improve the translation further.

Review of Cognitive Debriefing Result and Finalization

The findings of the cognitive debriefing session were reviewed again by the review committee. The findings and recommendations were analyzed, and amendments were made accordingly by the review committee.

Proofreading

The proofreading stage aimed to polish and consolidate the final version of the Malay-translated risk perception questionnaire. Any grammatical errors or typing errors were identified. Finally, the review committee took the final check for any further adjustments or corrections.

Final Report

A final report on the whole process was properly documented. Each correction and change applied was explained in words, as this was essential for future references.

Pre-testing

Before conducting the study, a pre-test was conducted to identify any potential issues with the translated questionnaire. This was done in one of the selected FELDA locations in Terengganu, with a group of 30 palm oil plantation workers recruited to participate. The pre-test was conducted face-to-face, and strict COVID-19 protocols were implemented during the session. Participants were given a hard copy of the questionnaire, and a briefing session was conducted beforehand to explain the purpose of the study and the pre-testing session. Feedback from participants indicated that some adjustments were needed, such as increasing the font size and adjusting the spacing between lines. These adjustments were made accordingly.

Data analysis

The data analysis for this study was conducted using IBM AMOS 26 software. The construct validity was assessed through confirmatory factor analysis (CFA) using the SEM tool in AMOS. The preferred analysis method was robust maximum likelihood. To determine the dimensionality of the scale, standardized factor loadings were calculated, with a factor loading of over 0.40 considered acceptable. The goodness of fit (GIF) for the model was evaluated using several criteria, including root mean square error of approximation (RMSEA), comparative fit index (CFI), and the chi-square test. The model was considered to have a relatively good fit if it met the following criteria: a CFI value greater than 0.90, a chi-square statistic divided by the degrees of freedom (df) value less than three, and a standardized root mean square residual (SRMR) and RMSEA values below 0.08, indicating adequate fit [17].

The model revision was undertaken based on factors such as factor loadings, standardized residuals (SR), modification indices (MI), and theoretical background. Parameters with SR values ≥|2.58| and MI values ≥3.84 were considered for potential changes in the model specifications. If a correlation of r was found to be ≥0.85, multicollinearity was anticipated.

In terms of reliability assessment, internal reliability consistency was determined using Cronbach's alpha, with a threshold of 0.7 or higher considered adequate [18].

## Results

The present study recruited a total of 385 participants, and all of them agreed to participate in the study before they were given the questionnaire. Only 381 of them completed the questionnaires giving a response rate of 99.0%. All of the participants were Malay, the majority of whom were male (82.7%). The mean (SD) age of the participants was 43.4 (16.28), with 11 years of average working experience. About 4.2% of the participants did not have any formal education, and a majority of the participants had monthly income between RM1001 to RM2000. More than half had experience working in other sectors (62.7%). Table 1 shows the characteristics of the participants of the study.

**Table 1:** Characteristics of the participants (n=381)

Characteristics (n=381)	n (%)	Mean (SD)
Gender		
Male	315 (82.7)	
Female	66 (17.3)	
Age (years)		43.4 (16.28)
Education level		
No formal education	16 (4.2)	
Had formal education	365 (95.8)	
Monthly income (RM)		
<1000	34 (8.9)	
1001-2000	172 (45.1)	
2001-3000	131 (34.4)	
>3000	44 (11.5)	
Working duration (years)		11.0 (10.00)
History of working in other sectors		
Yes	239 (62.7)	
No	142 (37.3)	

During the harmonization part of the translation process, the appointed expert panels reviewed all the items and agreed that all the items are relevant and adequately comprehensive. The item level of content validity index (CVI) (I-CVI) and scale level of CVI (S-CVI) both scored >0.8, which was considered good. The face validity and pilot testing revealed a few minor adjustments to wording preferences and the technical arrangement of the questionnaires. Otherwise, the instruction was simple and easily understood. Besides, using a five-point Likert Scale was considered appropriate and sufficient to capture the intended responses.

To select the best-fit model, there were four models discussed throughout the study. The initial model started with all the original 22 items. The majority of the items scored >0.60 except for four items, which had unacceptable standardized factor loading (item factor loading <0.41 or >1), specifically item 1 (“Do you think the tractor can overturn when working on sloping ground”), item 2 (“Do you consider vibrations (oscillations, shaking) in the agricultural sector relevant”), item 3 (“Do you think that gloves are useful for vibration protection”), and item 10 (“do you believe that jobs that require frequent and repeated use of upper limbs can cause disease”). After serial modification, eventually model 4 comprised of 13 items with satisfying factor loading (>0.6). Model 1 and model 2 did not achieve the standard value of model fit indices. Model 4 successfully achieved a reasonable fit for the Malay version of the Risk perception questionnaire indicated by CFA (X^2^=224, df=79, p-value<0.0001, RMSEA=0.07, GFI=0.929, CFI=0.902). Cronbach’s alpha for the 13-item questionnaire was α=0.77. Table 2 shows the model fit indexes of the questionnaire.

**Table 2:** Model fit indexes of the Malay version risk perception questionnaire X^2^, chi-square; df, degree of freedom; RMSEA, root mean square error of approximation; CFI, comparative fit index; GFI, goodness of fit index

Model	X^2^(df)	p-value	ChiSq/df	GFI	CFI	RMSEA
Model 1	1127 (209)	<0.0001	5.395	0.700	0.574	0.108
Model 2	734 (162)	<0.0001	4.586	0.831	0.715	0.097
Model 3	321 (107)	<0.0001	3.005	0.909	0.878	0.073
Model 4	224 (79)	<0.0001	2.842	0.929	0.902	0.070

## Discussion

This study aimed to translate and validate the original risk perception questionnaire developed by Professor Ceccini in central Italy for assessing risk perception among agricultural workers. The objective was to adapt the questionnaire to the Malay language and validate its psychometric properties. The final model of the translated questionnaire comprised 13 items, with nine items removed to achieve model fitness.

The translation and validation process of the risk perception questionnaire involved several important steps. First, a rigorous translation process was conducted to ensure semantic, conceptual, and cultural equivalence between the original and translated versions. The forward-backward translation method was employed, followed by a review by a panel of experts in the field of occupational health and safety to ensure content validity [13].

The translated questionnaire was then administered to a sample of agricultural workers in the state of Terengganu, Malaysia. The participants' responses were collected, and psychometric analyses were performed to evaluate the reliability and validity of the translated questionnaire. The internal consistency of the final 13-item model was assessed using Cronbach's alpha, which indicated satisfactory reliability (α=0.77) [18,19].

The removal of nine items from the original questionnaire was necessary to achieve model fitness during the validation process. These exclusions were based on statistical criteria, including factor loadings, item-total correlations, and conceptual clarity. While these nine items were deemed less relevant or redundant in the context of the Malaysian agricultural setting, the final 13-item model demonstrated acceptable psychometric properties and maintained the essence of the original questionnaire.

It is important to acknowledge the limitations of this study. First, the validation process was conducted in a specific geographic region (Terengganu, Malaysia) and focused on a specific occupational group (agricultural workers). Therefore, caution should be exercised when generalizing the findings to other populations or industries. Future research could further validate the translated questionnaire in different regions of Malaysia and among diverse agricultural worker populations to enhance its applicability and generalizability.

In conclusion, the translation and validation of the original risk perception questionnaire by Professor Ceccini to the Malay language provided a valuable tool for assessing risk perception among agricultural workers in Terengganu, Malaysia. The final 13-item model demonstrated satisfactory reliability and construct validity. This validated questionnaire can contribute to the measurement of risk perception and serve as a valuable instrument for future research and interventions aimed at enhancing occupational health and safety in the agricultural sector.

The findings of this study are expected to contribute significantly to the field of occupational health and safety in agriculture. By providing a validated risk perception questionnaire in the Malay language, this research will enable researchers and practitioners to assess and manage risks effectively in the agricultural sector. Additionally, the outcomes of this study will shed light on the specific risk perception factors and patterns unique to agricultural workers in Terengganu, facilitating the development of targeted interventions and policies to improve occupational health and safety outcomes in this region.

## Conclusions

In conclusion, the translation and validation of the original risk perception questionnaire by Professor Ceccini have resulted in a valid tool for assessing risk perception toward safety and health among agricultural workers. The process of translation and validation ensures the cultural and linguistic relevance of the questionnaire to be applied to Malay native speakers. The final model, consisting of 13 items, demonstrated good psychometric properties and achieved model fitness after the removal of nine items.
